# Mediastinal staging in non-small-cell lung carcinoma: computed tomography versus F-18-fluorodeoxyglucose positron-emission tomography and computed tomography

**DOI:** 10.1186/1470-7330-14-23

**Published:** 2014-06-03

**Authors:** Stefan Walbom Harders, Hans Henrik Madsen, Karin Hjorthaug, Anne Kirstine Arveschoug, Torben Riis Rasmussen, Peter Meldgaard, Johanne Andersen Hoejbjerg, Hans Kristian Pilegaard, Henrik Hager, Michael Rehling, Finn Rasmussen

**Affiliations:** 1 Department of Radiology, Aarhus University Hospital, Noerrebrogade 44, building 6, DK-8000 Aarhus, Denmark; 2 Department of Nuclear Medicine, Aarhus University Hospital, Aarhus, Denmark; 3 Department of Pulmonology, Aarhus University Hospital, Aarhus, Denmark; 4 Department of Oncology, Aarhus University Hospital, Aarhus, Denmark; 5 Department of Thoracic Surgery, Aarhus University Hospital, Aarhus, Denmark; 6 Department of Pathology, Aarhus University Hospital, Aarhus, Denmark

**Keywords:** Carcinoma, Non-small-cell lung/radiography, Carcinoma, Non-small-cell lung/radionuclide imaging, Mediastinal neoplasms/secondary, Computed tomography, X-ray/methods, Positron-emission tomography and computed tomography/methods, Sensitivity and specificity, Roc curve

## Abstract

**Background:**

After the diagnosis Non-Small-Cell Lung Carcinoma (*NSCLC*) has been established, consideration must turn toward the stage of disease, because this will impact directly on management and prognosis. Staging is used to predict survival and to guide the patient toward the most appropriate treatment regimen or clinical trial. Distinguishing malignant involvement of the mediastinal lymph nodes (N2 or N3) from the hilar lymph nodes, or no lymph nodes (N0 or N1) is critical, because malignant involvement of N2 or N3 lymph nodes usually indicates non**–**surgically resectable disease. The purpose of this study was to examine and compare CT versus integrated F18-FDG PET/low dose CT (*FDG PET*/*CT*) for mediastinal staging in NSCLC, and the desire was to safely distinguish between malignant and benign lesions without the need for invasive procedures. All results were controlled for reproducibility.

**Methods:**

114 participants with NSCLC were included in a prospective cohort study. Blinded CT and FDG PET/CT images were reviewed. The participants’ mediastinums were staged based on lymph node sizes (CT), or on FDG uptake (FDG PET/CT). Reference standard was tissue sampling.

**Results:**

We found that there was no measureable difference between CT and FDG PET/CT mediastinal staging results; overall two-thirds of the participants in the study were correctly staged, and almost one-third of the participants were falsely staged.

**Conclusion:**

Neither CT nor FDG PET/CT could obviate the need for further invasive staging prior to thoracotomy in patients with NSCLC; for that purpose, the results of both modalities were too meagre. Therefore, these patients still depend on invasive staging methods. In our study, invasive staging was accomplished by mediastinoscopy. However, today this is increasingly replaced by EBUS or EUS.

## Background

After a diagnosis Non-Small-Cell Lung Carcinoma (*NSCLC*) has been established, consideration must turn toward the stage of disease, because this will impact directly on management and prognosis. Staging is used to predict survival and to guide the patient toward the most appropriate treatment regimen or clinical trial. The most significant dividing line is between those patients who are candidates for surgery and those who may benefit from chemotherapy, radiation therapy, or both. The role of chemotherapy and radiation therapy followed by surgery for advanced disease is controversial [1]. Distinguishing malignant involvement of the ipsilateral or contralateral mediastinal lymph nodes (N2 or N3) from the ipsilateral hilar lymph nodes, or no lymph nodes (N0 or N1) is critical, because malignant involvement of N2 or N3 lymph nodes usually indicates non**–**surgically resectable disease.

CT is the most widely available and commonly used imaging modality for evaluation of the mediastinum in lung cancer. The majority of reports evaluating the accuracy of CT for mediastinal lymph node staging use a short-axis diameter of ≥ 1 cm on a transverse CT image as a threshold for abnormal nodes [1]. Using this threshold, the median sensitivity and specificity of CT for identifying mediastinal lymph node metastasis are 51%, and 86%, respectively [1]. These findings closely mirror a previous analysis on the matter by Gould et al. [2].

F-18-FDG PET (*FDG PET*) scanning has both higher sensitivity and higher specificity than CT scanning for the evaluation of mediastinal lymph nodes. This holds true even though there are no standardised quantitative criteria for abnormal FDG PET scan findings in the mediastinum [1]. Median estimates of sensitivity and specificity for identifying mediastinal lymph node metastasis are 74%, and 85%, respectively [1]. These findings are slightly less optimistic than those previously reported on the subject [2].

In some studies it has been claimed that the accuracy of FDG PET imaging in the mediastinum depends on the size of the nodes identified by CT. Indeed, Gould et al. reported median sensitivity and specificity of FDG PET scans of 100%, and 78%, respectively, in patients with enlarged lymph nodes [2]. Conversely, the median sensitivity and specificity of FDG PET scanning were 82%, and 93%, respectively, in patients with normal-sized lymph nodes [2].

However, even though FDG PET has been used for mediastinal staging for years, only few studies have compared CT and FDG PET/low dose CT (*FDG PET*/*CT*) with surgery as reference standard. Even fewer of these studies have examined the reproducibility of their results.

The purpose of this study was to examine and compare CT versus integrated FDG PET/CT for mediastinal staging in NSCLC, and the desire was to safely distinguish between malignant and benign lesions without the need for invasive procedures. This would have a significant diagnostic impact on patient management. The hypothesis was that FDG PET/CT was more sensitive and more specific than CT for staging the mediastinum. First, the overall mediastinal staging results of CT and FDG PET/CT were compared. Second, the staging results of FDG PET/CT were examined in patients with and without enlarged lymph nodes on CT. All results were controlled for reproducibility.

## Methods

### Ethics, consent

The study conformed to Danish legal requirements. As all subjects received best patient care and no biological material was involved, and as all images were entirely unidentifiable and there were no details on individuals reported within the manuscript, Institutional Review Board approval and individual informed consent for publication of images were waived (The Central Denmark Region Committees on Biomedical Research Ethics, case no. 1-15-0-72-2-09).

### Study population

Patients, who were recently diagnosed with NSCLC and were ready for staging, were prospectively identified for inclusion over a 2-year study period. All patients received CT as well as FDG PET/CT, and all metastasis suspect lesions were biopsied. Based on all available data, that is the CT, the FDG PET/CT and the biopsy results, a multidisciplinary staging was made: If the patients were staged with T1, N0, M0 disease, they received surgery. If the patients were staged with T2-T4, N0-N3, M0 disease, they received a preoperative mediastinoscopy; if they were eventually staged with T2-T4, N0-N1, M0 disease, they received surgery. In all other instances the patients received oncological treatment.

114 consecutive patients received CT as well as FDG PET/CT, and tissue sampling was obtained on all patients. Patient sampling preceded both imaging and reference standard. Therefore the study design was prospective.

### CT and FDG PET/CT procedures

As a part of a fast-track work-up for lung cancer, the patients received CT and FDG PET/CT within few days, immediately followed by tissue sampling.

CT included the chest and the upper abdomen. CT was performed with a multiple-row detector CT scanner (Philips Brilliance CT 64-channel scanner; Philips Healthcare, Best, the Netherlands). CT acquisition parameters were: 64 × 0.625 mm collimation. Reconstruction parameters were: 2.0 mm section thickness, and 1.0 mm increment. Iodixanole 270 mg/ml (Visipaque® 270; GE Healthcare, Oslo, Norway), or iohexole 300 mg/ml (Omnipaque® 300; GE Healthcare, Oslo, Norway), was injected intravenously in weight-adjusted doses of 2 ml/kg body weight to compensate for differences in distribution volume. A bolus tracking technique was used to compensate for differences in cardiac output. The trigger ROI was placed in the Aorta and when it exceeded 200 HU, the patients were scanned from the root of the neck to the upper abdomen including the liver and adrenals. CT was performed after a delay of 15 seconds for the chest, and 65 seconds for the upper abdomen, and raw picture data sets were transferred to a Philips Extended Brilliance™ Workspace workstation v4.02, where they were reviewed with the application CT-viewer.

**Figure 1:**
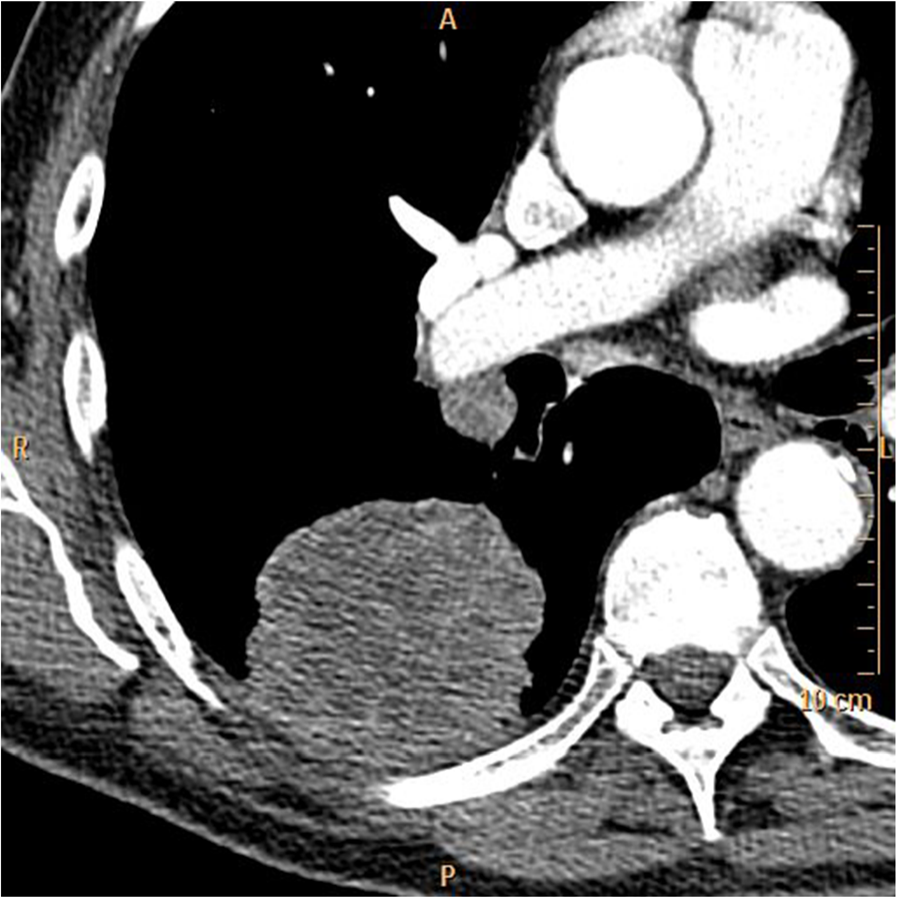
**This figure illustrates an enlarged ipsilateral hilar lymph node (N1 lymph node) in NSCLC.** The imaging modality is CT.

Two consultant radiologists reviewed the studies. The reviewers were blinded to patient name, patient ID and clinical data. Lymph nodes were characterised as normal-sized or enlarged; a short axis diameter ≥ 1 cm on a transverse CT scan was considered enlarged. Mediastinal staging was done on a per-patient basis, in accordance to the seventh edition of the TNM classification of malignant tumours [3] (Figure 1). Both radiologists reviewed all participants’ images side by side, to obtain consensus results for the study. Six months later, they reviewed the first 100 participants' images again, individually, to assess reproducibility.

Whole body FDG PET/CT including the head except for the brain, neck, thorax, abdomen, pelvis and thighs was performed with an integrated PET/CT scanner (Siemens Biograph w. 40-slice CT scanner; Siemens Healthcare, Erlangen, Germany). Participants were instructed to submit to 6 hours of fasting prior to the examination. Approximately 400 MBq FDG was injected intravenously. FDG PET/CT scans were performed after a delay of 60 minutes. The FDG PET images were corrected for scatter and iteratively reconstructed. CT acquisition parameters were: 40 × 3.0 mm collimation. Reconstruction parameters were: 5.0 mm section thickness, and 3.0 mm increment. No contrast medium was administered. FDG PET/CT picture data sets were transferred to a Hermes Gold 3™ workstation, where they were reviewed with the application Hermes Hybrid Viewer.

**Figure 2:**
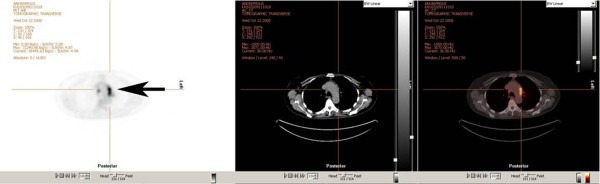
**This figure illustrates definitely increased FDG uptake of ipsilateral mediastinal and aortic lymph nodes (both, N2 lymph nodes) in NSCLC.** The imaging modality is FDG PET/CT.

Two consultants in nuclear medicine did the FDG PET/CT reviews. The reviewers were blinded to patient names, patient IDs and clinical data. According to international guidelines [4,5], FDG uptake was compared to the background uptake of the liver. Thus, lymph node uptake was rated on a scale of 1 to 3: 1) no uptake, 2) probably increased uptake and 3) definitely increased uptake. A rating of 1 was considered normal, a rating of 2 or 3 was considered abnormal. Mediastinal staging was done on a per-patient basis, in accordance to the seventh edition of the TNM classification of malignant tumours [3] (Figure 2). Low dose CT images were used for attenuation correction, lesion location and measuring purposes only. Both nuclear medicine consultants reviewed all participants’ images side by side to obtain consensus results for the study. Six months later, they reviewed the first 100 participants’ images again, individually, to assess reproducibility.

The radiologists had no access to FDG PET/CT images, and the nuclear medicine physicians had no access to CT images. Thus, the reviewers were completely blinded.

### Reference standard

Tissue sampling from the patients’ mediastinums was the reference standard in this study. In patients who did not receive surgery, tissue sampling was obtained by preoperative mediastinoscopy with sampling from nodal stations 1, 2R/L, 3A, 4R/L, and 7; if necessary, it was obtained by anterior mediastinotomy from nodal stations 5, and 6. All mediastinoscopies/-tomies were guided by both CT and FDG PET/CT examinations. In patients, who received surgery, tissue sampling was obtained by complete lymph node resection (i.e. resection of all visible and palpable mediastinal and hilar lymph nodes from nodal stations 2R, 4R, 7, 8, 9, 10, 11+ for right-sided tumours, and 5, 6, 7, 8, 9, 10, 11+ for left-sided tumours).

### Statistics

According to the significance of involvement of N2 or N3 lymph nodes, all participants were classified as having either positive (N2 or N3) or negative (N0 or N1) staging results and as having or not having mediastinal lymph node involvement as determined by the reference standard. Three statistical methods were used to describe the data:

First, diagnostic accuracy was defined as the area under the parametric Receiver Operating Characteristic (*ROC*) curve, and was computed using the ratings with a maximum-likelihood ROC model assuming bivariate normal distributions [6].

Second, sensitivity, specificity, positive predictive value, and negative predictive value were computed from the resulting 2×2 contingency tables. False positive rate (1 – specificity), and false negative rate (1 – sensitivity) were also computed.

Third, the reproducibility of the results was assessed with weighted kappa of the original ratings.

Sample test statistics was used when appropriate; Spearman’s rho was used to test for correlation between ordered categorised (ordinal) variables. The chi-square test was used to test for non-independence of the areas under the ROC curves of CT and FDG PET/CT. P-values < 0.05 were considered statistically significant, and p-values < 0.001 were considered highly statistically significant.

The licensed statistical software package STATA/SE 11 (STATAcorp LP, College Station, Texas, United States), was used.

## Results

### Overall results

114 patients participated in the study. 89 of these patients received surgery with complete lymph node resection; 25 received mediastinoscopy/-tomy only. The nodal stage distribution was 61% (69/114) N0, 13% (15/114) N1, 23% (26/114) N2, and 4% (4/114) N3.

**Figure 3:**
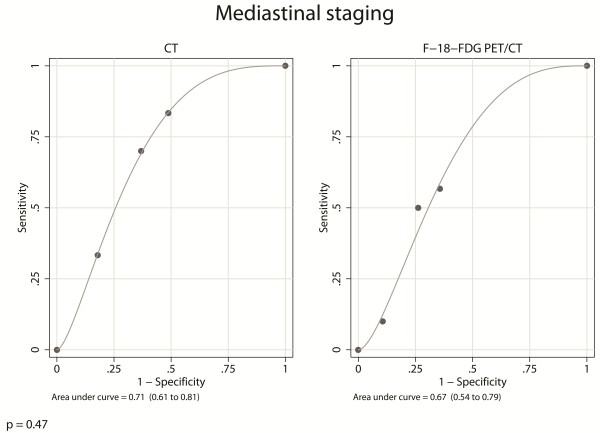
**These two parametric ROC curves illustrate the overall mediastinal staging results of CT and FDG PET/CT.** In this study the overall diagnostic accuracy of CT and of FDG PET/CT was defined as the area under the parametric ROC curves. The two Roc curves were compared using the chi-square test.

There was no significant difference between the areas under the ROC curves of CT and F- 18-FDG PET/CT (χ^2^ = 0.53; p = 0.47) (Figure 3). However, whereas CT results were significantly associated to reference standard results (ρ = 0.29; p = 0.002), FDG PET/CT results were not (ρ = 0.16; p = 0.08).

CT and FDG PET/CT ratings are presented in wide layout below (Table 1).

**Table 1:** CT and FDG PET/CT ratings

***Reference std*. *N0*/*N1***	**N0 (CT)**	**N1 (CT)**	**N2 (CT)**	**N3 (CT)**	**Total**
N0 (FDG PET/CT)	31	7	8	8	54
N1 (FDG PET/CT)	5	2	0	1	8
N2 (FDG PET/CT)	3	1	7	2	13
N3 (FDG PET/CT)	4	0	1	4	9
Total	43	10	16	15	84
***Reference std. N2/N3***	**N0 (CT)**	**N1 (CT)**	**N2 (CT)**	**N3 (CT)**	**Total**
N0 (FDG PET/CT)	4	3	3	3	13
N1 (FDG PET/CT)	1	0	1	0	2
N2 (FDG PET/CT)	0	1	7	4	12
N3 (FDG PET/CT)	0	0	0	3	3
Total	5	4	11	10	30

Paired CT (columns) and FDG PET/CT (rows) ratings when the reference std. was either N0/N1 or N2/N3.

CT correctly classified 65% (74/114) of the cases; 27% (31/114) were falsely overstaged, and 7% (9/114) were falsely understaged. The overall sensitivity and specificity of CT for mediastinal staging were 70%, and 63%, respectively (Table 2). Reproducibility of the CT stagings was substantial (κ = 0.63 (0.47 to 0.77)).

**Table 2:** Overall results of CT and FDG PET/CT (n = 114)

	**CT**	**FDG PET/CT**
Sensitivity	70% (51% to 85%)	50% (31% to 69%)
Specificity	63% (52% to 73%)	74% (63% to 83%)
Positive predictive value	40% (27% to 55%)	41% (25% to 58%)
Negative predictive value	86% (74% to 93%)	81% (70% to 89%)
False positive rate	37%	26%
False negative rate	30%	50%

Derived from Table 1. Results are presented as estimates with 95% confidence intervals.

FDG PET/CT correctly classified 68% (77/114) of the cases; 19% (22/114) were falsely overstaged, and 13% (15/114) were falsely understaged. The overall sensitivity and specificity of FDG PET/CT for mediastinal staging were 50%, and 74%, respectively (Table 2). Reproducibility of the FDG PET/CT stagings was moderate (κ = 0.57 (0.39 to 0.72)).

The false positive rates of CT and FDG PET/CT were 37%, and 26%, respectively; the false negative rates were 30%, and 50%, respectively. The false negative lymph nodes were located to stations 3A, 4R/L, and 7; this was irrespective of imaging modality.

### Stratified results

Next, CT results were used to stratify FDG PET/CT results into subgroups.

In patients with enlarged lymph nodes on CT (n = 52), the sensitivity and specificity of FDG PET/CT were 67%, and 55%, respectively; the false positive rate was 45% (Table 3).

**Table 3:** Stratified results of FDG PET/CT, in patients

	**With enlarged lymph nodes (n = 52)**	**Without enlarged lymph nodes (n = 62)**
Sensitivity	67% (43% to 85%)	11% (0% to 48%)
Specificity	55% (36% to 73%)	85% (72% to 93%)
Positive predictive value	50% (31% to 69%)	11% (0% to 48%)
Negative predictive value	71% (49% to 87%)	85% (72% to 93%)
False positive rate	45%	15%
False negative rate	33%	89%

Derived from Table 1. Results are presented as estimates with 95% confidence intervals.

In patients without enlarged lymph nodes on CT (n = 62), the sensitivity and specificity of FDG PET/CT were 11%, and 85%, respectively; the false negative rate was 89% (Table 3).

## Discussion

The aim of this study was to examine and compare CT versus integrated FDG PET/CT for mediastinal staging in NSCLC, and the desire was to safely distinguish between malignant and benign lesions without the need for invasive procedures. The hypothesis was that FDG PET/CT was more sensitive and more specific than CT for staging the mediastinum. For this purpose, 114 participants with NSCLC were included in a prospective cohort study. Blinded CT and FDG PET/CT images were reviewed. The participants’ mediastinums were staged based on lymph node sizes (CT), or on FDG uptake (FDG PET/CT). Reference standard was tissue sampling. Overall diagnostic accuracy was defined as the area under the ROC curve, and sensitivity, specificity, positive predictive value, and negative predictive value were calculated. Reproducibility was measured with kappa statistics.

The overall prevalence of N2 or N3 disease was 26%. Overall, there was no significant difference between CT and FDG PET/CT staging results. As such, approximately two-thirds of the patients were correctly staged by using either CT or FDG PET/CT; the remaining one-third of the patients was incorrectly staged. Whereas CT tended to overstage the mediastinal nodes, FDG PET/CT tended to both overstage and understage these. The sensitivity and specificity of CT were 70%, and 63%, respectively, and the sensitivity and specificity of FDG PET/CT were 50%, and 74%, respectively. When the false positives and false negatives were reviewed, it was noteworthy that whereas the false positive rates of both CT and FDG PET/CT were approximately 30% to 33%, the false negative rate of FDG PET/CT was as high as 50%. Based on these results two important messages emerged. First, approximately one-third of all benign nodes are falsely deemed to be malignant, irrespective of the imaging modality. Second, as many as half of all malignant nodes are falsely deemed to be benign on FDG PET/CT. Thus, both imaging modalities can both overstage and understage the mediastinal nodes.

Next, the presence of enlarged lymph nodes on CT was used to stratify FDG PET/CT results into subgroups. If there were enlarged lymph nodes on CT, the number of true positives (sensitivity) and false positives of FDG PET/CT increased. Thus, in patients with enlarged lymph nodes, FDG PET/CT is more likely to reveal both true positive findings that are due to lymph node metastasis and false positive findings that are due to infection or inflammation, respectively. However, because the negative consequences of false positives are so serious, a positive FDG PET/CT should not automatically “rule in” N2 or N3 disease, and these patients should receive a mediastinoscopy, unless distant metastases were proven beforehand. Failure to do so could result in patients with surgically resectable disease being denied curative surgery. In fact, in that context, an FDG PET/CT examination would make no difference to this group of patients. Conversely, if there were no enlarged lymph nodes on CT, the number of true negatives (specificity) of FDG PET/CT rose substantially, but there was excessive 89% false negatives! Whether a negative FDG PET/CT would obviate the need for mediastinoscopy in these patients has been controversial, though according to our results, it would not. In that context, an FDG PET/CT examination would not make any difference to these patients either.

These results are somewhat controversial, considering that the general agreement is that FDG PET is both highly accurate for mediastinal staging of NSCLC, and that it is superior to CT. In two large meta-analyses of articles on the matter published since the mid 1990s [1,2], it is claimed more than once that FDG PET has both sensitivity and specificity for mediastinal staging of NSCLC of almost 90%. Although the overall prevalence of N2 and N3 disease of our study (26%) is almost exactly identical to the prevalence in these meta-analyses (29% and 32%, respectively), we are nowhere near their results. Most likely some or all of the studies included in the meta-analyses represent highly selected study participants whereas our study is set in our everyday clinical population. Thus, our results seem to be more related to the results from some newer studies on the matter [7-12].

There are some limitations to our study, the most important being whether all mediastinal lymph node metastases were detected by the reference standard. Especially in the patients that were only examined with mediastinoscopy/-tomy this could be an issue. The diagnostic yield of mediastinoscopy/-tomy is operator dependent, and the false negative rate is estimated to be between 3% and 20% [1]. However, considering that these procedures were only used to confirm N2 or N3 disease, they were accepted as reference standard.

Since this study was carried out, mediastinoscopy has been replaced by Endobronchial Ultrasound with Transbronchial Needle Aspiration (*EBUS*-*TBNA*) and Endoscopic Ultrasound with Needle Aspiration (*EUS*-*FNA*), due to these methods’ attractive combination of high diagnostic yield and low risk. EBUS-TBNA is a relatively new technique for mediastinal staging, which can be performed on an outpatient basis. EBUS-TBNA can be used to sample nodal stations 1, 2R/L, 4R/L, 7, 10R/L, 11R/L and 12R/L. In eight studies of EBUS-TBNA of the mediastinum, the sensitivity was 79% to 95% (median, 90%) and the false negative rate was 1% to 37% (median, 24%) [13]. EUS-FNA of mediastinal lymph nodes through the wall of the oesophagus can also be performed on an outpatient basis. No mortality has been reported. EUS-FNA is particularly useful for nodal stations 4 L, 5, 7, 8 and 9. In 16 studies of EUS-FNA of the mediastinum, the sensitivity was 45% to 100% (median, 84%) and the FNR was 0% to 61% (median, 19%) for the detection of N2 or N3 malignant mediastinal lymph nodes [13].

Conversely some important strength to our study must be mentioned. First, patient sampling preceded both imaging and reference standard. This prospective design is a strength, as well as the blinding procedure, as the STARD statement from 2003 dictates that these are both natural requisites in studies of diagnostic accuracy [14]. Furthermore, there is the large study size: 114 participants with NSCLC were included, and all participants were examined with both CT and with FDG PET/CT. This should be compared to an average study size of 118 participants for CT, and 65 participants for FDG PET [1], and 51 participants for both CT and FDG PET [2], making this study comparably strong. Ultimately, all results in this study were controlled for reproducibility. Though this has been standard for CT since the STARD statement, to the best of our knowledge, reproducibility has not previously been controlled for FDG PET/CT.

## Conclusions

In conclusion, controversially, CT was superior to FDG PET/CT for mediastinal staging of NSCLC in more ways than one. We found that the sensitivity of CT was higher than for FDG PET/CT, and more importantly, that the number of false negatives of CT was lower than for FDG PET/CT. However, we also found that neither CT nor FDG PET/CT could obviate the need for further invasive staging prior to thoracotomy in patients with NSCLC; for that purpose, the results of both modalities were too meagre. Finally, we found that FDG PET/CT was not clinically feasible for staging the mediastinum - regardless of whether the patients had or did not have enlarged lymph nodes on CT.

An important clinical perspective of this study is that, according to our results, neither CT nor FDG PET/CT, individually or in combination, can obviate the need for invasive staging prior to thoracotomy. This is regardless of whether the patients to be staged are unselected or if they are initially CT scanned, and then FDG PET/CT scanned. There are too many false positives and especially too many false negatives. Therefore, these patients still depend on invasive staging methods. In our study, invasive staging was accomplished by mediastinoscopy. However, today this is increasingly replaced by EBUS or EUS [13].

## Competing interests

The authors declare that they have no competing interests.

## Authors’ contributions

SWH, HHM, FR, KH, AKA and MR were involved in the conception and design of the study. TRR included the participants, HKP performed the surgical procedures and HH analysed the pathological specimens. SWH created the dataset. SWH, HHM, FR, KH, AKA and MR performed the analysis and data interpretation. SWH drafted the article. All authors were involved in the critical revision of the manuscript and gave final approval of the version submitted. SWH is guarantor.
